# Access to pediatric rheumatology care for Juvenile Idiopathic Arthritis in the United Arab Emirates

**DOI:** 10.1186/s12969-017-0170-4

**Published:** 2017-05-16

**Authors:** Khulood Khawaja, Mustafa Al-Maini

**Affiliations:** Department of Rheumatology, Allergy and Immunology, Al-Mafraq Hospital, P.O. Box 2951, Abu Dhabi, United Arab Emirates

**Keywords:** Juvenile Idiopathic Arthritis, Access, Care, Pediatric rheumatology

## Abstract

**Background:**

This study looks at access to care for Juvenile Idiopathic Arthritis through pediatric rheumatology in the UAE, as an example of multi-ethnic society.

**Methods:**

Patients with a diagnosis of Juvenile idiopathic arthritis were identified through the hospital electronic medical records system from January 1st 2011 to December 31st 2014. All residents of the United Arab Emirates hold an Emirates identity card. We divided our patients into two groups: Emirati-Emirates, who are native Emirati children and hold the Emirati nationality, as stated on their Emirates identity card, and who therefore have full, comprehensive access to free medical care; and non-Emirati-Emirates, who represent other nationalities, as stated on their Emirates identity card. The primary objective of this study is to look at access to care for Juvenile idiopathic arthritis through pediatric rheumatology in the two groups. The secondary objective is to look at the effect of having multiple types of healthcare insurance coverage on access to biologics. A retrospective review was carried out.

**Results:**

Sixty-six patients with JIA identified: 33 Emirates and 33 non-Emirates. For Emirates, the mean time from onset to first appointment with pediatric rheumatologist and diagnosis is 9 months (range: 1–48), and for non-Emirates is 12.4 months (range: 1–96). Among the Emirates, 10 patients are currently on biologic with methotrexate. Among the non-Emirates, 15 are on biologic with methotrexate. Among the Emirates, 12 are currently in remission while on treatment, as are 10 non-Emirates. Regarding disability, one Emirati patient has blindness secondary to noncompliance while under previous treatment. One Non-Emirati developed joint deformities due to periods of noncompliance and no follow up.

**Conclusions:**

Delay in presentation to pediatric rheumatology has been identified as an important factor in our population, which is multi-cultural and multi-ethnic. Type of health care insurance cover did not affect number of patients getting biological therapy once patient seen in the pediatric rheumatology service.

## Background

Juvenile Idiopathic Arthritis (JIA) is a chronic inflammatory disease that affects 1 in 1000 children worldwide [1]. It is a heterogeneous group of autoimmune conditions classified into seven different subtypes, based on the clinical features in the first 6 months of presentation, according to International League of Associations of Rheumatology’ classification system [2]. UAE’s population of 9,346,129 [3] is diverse: 19% of residents are Emirati-Emiratis (EE), who are native Emiratis, which means that they were born in the UAE and originate from the UAE; 81% are non-Emirati-Emiratis (non-EE), who were born in the UAE or other countries and who are originally from outside the UAE, according to their Emirates identity card. The non-EE population includes 23% other Arabs, 50% South Asians, and 8% other expatriates, including Westerners and East Asians [4]. Abu-Dhabi is the capital city of the UAE with a population of 2.3 million [3]. The tertiary Pediatric rheumatology centre is in one of the two main public hospitals in Abu-Dhabi. An out reach clinic provided once weekly to the other main public hospital in Abu Dhabi. Patients included from both hospitals as the can be seen in either hospital.

There is limited access to pediatric rheumatology care worldwide, and this is particularly true in the United Arab Emirates (UAE). A recent publication by the World Forum on Rheumatic and Musculoskeletal Diseases identified a severe shortage of pediatric rheumatologists both in developed and developing countries [5].

There are also differences in healthcare insurance coverage within the UAE population. It is premium-free and comprehensive for EE children with easy access to public hospitals. For non-EE children health cover is variable from basic emergency cover to full comprehensive treatment. Access to the public hospitals is more difficult for the non-EE due to type of health care insurance cover.

Non-EE child can be referred through “public” primary health care clinic if covered by health insurance or through private sector as pediatric rheumatology considered unique service since it is not available in the private sector. The insurance company still needs to approve the service and treatment. If this is not possible there are government schemes available to fund such treatments once the child seen by the specialist and comprehensive justification for treatment reported.

Our tertiary pediatric rheumatology service is available in the two main public hospitals in the capital city of Abu-Dhabi. The centre is based in one hospital and a weekly outreach clinic is provided in the other public hospital. Referrals are accepted from public and private healthcare providers throughout the country.

The primary objective of this study is to look at access to care for JIA through pediatric rheumatology in the UAE, which is taken as an example of multi-ethnic society. The secondary objective is to look at effect of multiple types of healthcare insurance coverage on access to biologics.

## Methods

Patients with a diagnosis of JIA were identified through the hospital electronic medical records system (EMR), which was implemented for all medical documentation in January 2011. Using the International Statistical Classification of Diseases and Related Health Problems (usually referred to by the shorter name “International Classification of Diseases (ICD)), version 9 (ICD 9) (ICD 9 code 714, 714.30,714.89, 696) from January 1st 2011 to December 31st 2014, we sought out all patients under 16 years of age with a diagnosis of JIA. A retrospective review of the electronic medical records (EMR) and paper case notes of the patients who presented to the two main public hospitals in Abu-Dhabi providing tertiary pediatric rheumatology service.

All residents of the UAE hold an Emirates identity card. We divided our patients into two groups: Emirati-Emirates (EE), who are native Emirati children and hold the Emirati nationality, as stated on their Emirates identity card, and who therefore have full, comprehensive access to free medical care; and non-Emirati-Emiratis (non-EE), who represent other nationalities, as stated on their Emirates identity card. Non-EEs can have variable medical insurance coverage as explained in the background.

For the primary objective to look at access to care for JIA through pediatric rheumatology in the UAE we examined the following: percentage of EE children relative to total number of children; subtypes of JIA, according to International League of Associations for Rheumatology (ILAR) classification in each group [2]; time in months (with ranges) from onset to first appointment in pediatric rheumatology and diagnosis, number of health professionals seen prior to referral to pediatric rheumatology, number of self referrals; number of patients on steroids prior to first appointment with rheumatology, number of patients referred to ophthalmology and number of patients with uveitis.

To assess effect of health care insurance coverage on access to biologics we looked at the number of patients on disease-modifying antirheumatic drugs (DMARD) and number of patients on biologics in each group.; number of patients in remission on treatment “Clinical remission on treatment” is defined according to American College of Rheumatology of clinically inactive disease for at least six continuous months while on therapy. (Symptom free, have a CHAQ score of 0, joint count of 0, and normal inflammatory markers). Also number of patients with a disability secondary to JIA.

The information was recorded in a case report form (CRF). Ethical approval was obtained through the hospital’s ethics review board.

### Data analysis

Data from CRF were entered into a database (Microsoft Office Excel, Microsoft, USA). Descriptive analysis was then performed.

## Results

In total, 66 cases with JIA are identified: 33 EE (26 female, 7 male) and 33 non-EE (21 female, 12 male), as seen in Table 1. Of the 33 EEs, the following subtypes of JIA are found: 4 systemic, 10 persistent oligoarticular, 2 extended oligoarticular, 17 polyarticular (7 Rheumatoid factor (RF) + ve, 10 RF-ve).

**Table 1:** Patient Characteristics

	Emirati-Emiratis	Non-Emirati-Emiratis
Total number	33	33
Female/male	26/7	21/12
Systemic	4 (12.2%)	9 (27.3%)
Persistent Oligo JIA	10 (30.3%)	6 (18.2%)
Extended Oligo JIA	2 (6%)	1 (3%)
Poly JIA RF-ve	10 (30.3%)	10 (30.3%)
Poly JIA RF + ve	7 (21.2%)	5 (15.2%)
Enthesitis-related JIA	-	1 (3%)
Psoriatic	-	1 (3%)
Mean time from onset to first appointment with Ped Rheum (months)	9	12.4
	Range: 1–48	Range: 1–96
Median time from onset to first appointment with Pediatric rheumatology	10 months	14 months
Oral steroids at diagnosis	31 (94%)	32 (97%)
>2 health professionals seen before pediatric rheumatologist	31 (94%)	25 (75.7%)
Referral to ophthalmology prior to pediatric rheumatology	7 (21.2%)	7 (21.2%)
Uveitis	3 (9%)	2 (6%)
MTX alone	10 (30.3%)	5 (15.2%)
Biologic + MTX treatment	10 (30.3%)	15 (45.5%)
Remission on treatment	12 (36.3%)	10 (30.3%)
Disability	1 blind (uveitis)	1 multiple joint deformities

*JIA* Juvenile Idiopathic Arthritis, *MTX* Methotrexate

Among the 33 non-EE cases, the subtypes include 9 systemic, 6 persistent oligoarticular, 1 extended oligoarticular, 15 polyarticular (5 RF + ve, 10 RF-ve), 1 enthesitis related arthritis, 1 psoriatic arthritis).

For EEs, the mean time from onset to first appointment with a pediatric rheumatologist and diagnosis is 9 months, ranging from 1 to 48, while for non-EEs it is 12.4 months, ranging from 1 to 96. Of the 33 EEs, 31 (94%) saw more than two health professionals prior to referral to pediatric rheumatology, as did 25 (75.7%) of the 33 non-EEs.

In each subgroup, 25 patients (75%) had seen two professionals before getting an appointment with a pediatric rheumatologist; 80% saw a pediatrician, 70% an adult rheumatologist, and 60% an orthopedic surgeon. Only 30% of all patients were referred with a possible diagnosis of JIA; the rest were referred with swollen joints query cause or deteriorating function.

For EEs, 30 of the 33 (91%), and for non-EEs, 32 of the 33 (97%), including the persistent oligoarticular subgroup, have received oral steroids.

With regards to uveitis screening, 10 EEs and 7 non-EEs have documented referral to ophthalmology.

Among the EE patients, 15 (45%) of the 33 were self-referrals, as were 13 (39%) of the 33 non-EEs. There was minimum of 4 years of follow up for all patients.

Among the EEs, 10 patients are currently on biologic with methotrexate (4 etanercept, 2 adalimumab, 1 infliximab, 2 tocilizumab, and 1 abatacept), and 10 are on methotrexate alone. Among the non-EEs, 15 are on biologic with methotrexate (7 etanercept, 2 adalimumab, 1 infliximab, and 5 tocilizumab), and 5 are on methotrexate alone.

In each group, 12 are currently in remission on treatment.

Regarding disability, One EE patient with blindness due to periods of non-compliance is under treatment for uveitis. The patient was lost to follow-up for extended periods of time, then returned with complete loss of vision in one eye and limited vision in the other eye.

The non-EE with multiple joint deformities due to periods of non-compliance also had avascular necrosis of one of his hips. He was on long-term oral steroids when he presented to pediatric rheumatology.

## Discussion

There are little published data from developing countries on JIA patients’ access to tertiary pediatric rheumatology care. Long delays after the onset of JIA in starting definitive treatment remain an important factor, and this is likely to affect the outcome of JIA patients in our region, as is the case in other parts of the world [6–8].

Western literature and one study from Costa Rica offer evidence regarding delays in accessing pediatric rheumatology [9–18] but there is no data available from our country on accessing pediatric rheumatology service.

Our results showed delay for accessing pediatric rheumatology service for both EE and non-EE. Our numbers are small; this in itself reflects the under-referral of JIA patients. According to the population statistics, we should have had more non-EE children presenting with JIA and more South Asian families. For non-EE patients, the type of health insurance coverage might have influenced this and the longer duration of delay in referral compared to EE patients. Patients might have had to go to the private sector because their insurance did not cover care in the public sector. The British Society of Pediatric and Adolescent Rheumatology stressed the importance of early recognition of JIA and prompt referral, with a target of 2 weeks from disease onset to the first appointment in pediatric rheumatology, as this would improve outcomes among JIA patients [19].

There is still lack of awareness among health professionals of the presentations of JIA, hence the lengthy referral time to pediatric rheumatology, lack of referral to opthalmology and multiple professionals assessing the child prior to referral. We know from documented literature in the west that doctors do not feel confident in carrying out musculoskeletal examination [20, 21], as pediatric rheumatology is not part of the core curriculum in most of medical schools [22]. Children with JIA usually present to general pediatricians, orthopedic doctors, and adult rheumatologists, who rely on their experience and teaching during medical school.

We carried out a survey of our group of 40 pediatric residents in training in the two teaching hospitals in the capital city prior to delivering musculoskeletal (MSK) teaching session. The results showed that 39 of the 40 residents did not have a method for MSK screening examination in children and did not feel confident in screening the musculoskeletal system in children presenting with joint pain “unpublished data”.

Geographical factors could have been a reason for the delay of presentation for some of our patients. The UAE spans over 83,600 km^2^, with a population of 9,346,129 [4], and is made up of seven emirates.

There is a visiting consultant once a month in the private sector and an experienced pediatrician in the public sector providing pediatric rheumatology in other area of the country. This could have partially influenced the results.

We identified more non-EE children than EE children presenting with systemic subtype who were acutely unwell. Non-EE children are more likely to come to the hospital if they are acutely unwell, and they tend to wait longer if presenting with other subtypes of JIA with a chronic history. This has been reported previously in the literature for oligoarticular disease [23], and may explain why we only had six persistent oligoarticular JIA cases in the non-EE group and an equal number of polyarticular JIA in both groups. Oligoarticular patients are not acutely unwell, and non-EE patients dependent on healthcare insurance coverage are more likely to present to private healthcare clinics.

We believe that public awareness is important in our population, as 45% of EE and 39% of non-EE initial appointments were a result of self-referral this can be partly explained by socioeconomic factors. It has been previously reported in the literature that patients from South Asian communities and with a higher level of education are more likely to present early, reflecting the parents’ empowerment [24, 25]. However, the majority of non-EE children’s parents are migrant workers of less privileged socioeconomic backgrounds, and these families opted to self-refer for their children to get appropriate treatment. More EE patients came through self-referral, which reflects easier access to public sector compared to non-EE patients.

There was significant over use of oral steroids in both groups prior to referral to pediatric rheumatology, including oligoarticular patients, as there was limited access to joint injections with steroids under general anesthetic.

Type of health care insurance cover did not affect type of treatment provided as 10 out of 33 EE patients and 15 out of 33 non-EE patients received biological therapy. This could be due to the fact that we had more systemic and less oligoarticular non-EE patients. For EE patients the government funds biological therapy. For non-EE patients the funding is through insurance cover. There are also government schemes, which can be obtained for children needing biologics with inadequate health insurance cover. However, we believe our numbers might not be a true reflection of the overall care of children needing biologics, as our numbers for non-EE children are lower than what is expected for our population.

We identified two cases with disability, one in each group. The child with blindness had periods of poor compliance and failure to follow up while under treatment. The other child also had periods of non-compliance and sought alternative therapy with over-use of steroids, resulting in avascular necrosis of the hip. The rate of disability is probably underrepresented, in view of the retrospective nature of this work. We found that most children with only uveitis are looked after by ophthalmologists alone, and referral rates from orthopedics to pediatric rheumatology are low. We are conducting two separate studies to look at these issues.

Education is one important aspect to improve access to pediatric rheumatology care over the next 10 years in the UAE. This can possibly be achieved by incorporating musculoskeletal examination in to the core curriculum of our medical students. Foster’s pediatric Gait, Arms, Leg and Spine exam is a simple tool that can be used [26]. Also increasing education for resident trainees on musculoskeletal examination, JIA and other rheumatic conditions is needed. Developing postgraduate courses for family doctors, pediatricians, emergency doctors and orthopedic surgeons would potentially improve access to care and reduce delay in referrals. Following these changes our study can be repeated to access improvement of access to care in the UAE.

Comprehensive care for our patients should be provided through multidisciplinary networks, including both public and private, to address all aspects of care for our children with arthritis.

## Conclusions

Delay in presentation to pediatric rheumatology have been identified as an important factor in our population, which is multi-cultural and which represents many socioeconomic backgrounds. The reasons for delays are likely multi-factorial. Access to care, lack of awareness, clinical skills, professional networks, and geographical factors all may play a role. Type of health care insurance cover did not affect number of patients getting biological therapy once patient seen in the pediatric rheumatology service. We are currently working with other healthcare providers to address access to care.

## Abbreviations

ACR: American College of Rheumatology
CHAQ: Child Health Assessment Questionnaire
CRF: Clinical research form
DMARD: Disease-modifying antirheumatic drug
EE: Emirati-Emiratis (holds Emirati nationality, as stated on Emirates identity card)
EMR: Electronic medical records
ICD: International Statistical Classification of Diseases and Related Health Problems
ILAR: International League of Associations for Rheumatology
JIA: Juvenile Idiopathic Arthritis
MSK: Musculoskeletal system
Non-EE: Nationality other than Emiratis stated on Emirates identity card
RF: Rheumatoid factor
UAE: United Arab Emirates
